# Committees Appointed by the American Medical Association, for 1853–4

**Published:** 1853-08

**Authors:** 


### ECLECTIC DEPARTMENT,

### AND SPIRIT OF THE MEDICAL PERIODICAL PRESS.

---

*Committees appointed by the American Medical Association, for* 1853–4. The Committee on Nominations, in fulfilling the duty of their appointment, propose to continue most of the Special Committees appointed by the Association, in May, 1851, and May, 1852, and to appoint several new Special Committees. They, therefore, submit the following list of Chairmen for Special Committees, with the subjects to them committed:

1. Dr. D. F. Condie, of Philadelphia, Penn., "On the Causes of Tubercular Disease."
2. Dr. James Jones, of New Orleans, La., "On the Mutual Relations of Yellow and Bilious Remittent Fever."
3. Dr. R. S. Holmes, of St. Louis, Mo., "On Epidemic Erysipelas."
4. Dr. George B. Wood, of Philadelphia, Penn., "On Diseases of Parasitic Origin."
5. Dr. R. D. Arnold, of Savannah, Ga., "On the Physiological Peculiarities and Diseases of Negroes."
6. Dr. James R. Wood, of New York, "On Statistics of the Operation for the Removal of Stone in the Bladder."
7. Dr. F. Peyre Porcher, of Charleston, S. C., "On the Toxicological and Medicinal Properties of our Cryptogamic Plants."
8. Dr. Goodrich A. Wilson, of Virginia, "On Cholera and its Relation to Congestive Fever—their Analogy or Identity."
9. Dr. Worthington Hooker, of Connecticut, "On Epidemics of New England and New York."
10. Dr. John L. Atlee, of Lancaster, Penn., "On Epidemics of New Jersey, Pennsylvania, Delaware and Maryland."
11. Dr. D. J. Cain, of Charleston, S. C., "On Epidemics of South Carolina, Florida, Georgia and Alabama."
12. Dr. W. L. Sutten, of Georgetown, Ky., "On Epidemics of Tennessee and Kentucky."
13. Dr. Thomas Reyburn, of St. Louis, Mo., "On Epidemics of Missouri, Illinois, Iowa and Wisconsin."
14. Dr. George Mendenhall, of Cincinnati, Ohio, "On Epidemics of Ohio, Indiana and Michigan."
15. Dr. E. D. Fenner, of New Orleans, La., "On Epidemics of Mississippi, Louisiana, Texas and Arkansas."
16. Dr. Charles A. Lee, of New York, "On Domestic Hygiene."
17. Dr. Daniel Brainard, of Chicago, Ill., "On the Constitutional and Local Treatment of Carcinoma."

18. Dr. N. S. Davis, of Chicago, Ill., "On the Influence of Local Circumstances on the Origin and Prevalence of Typhoid Fever."

19. Dr. Geo. Engelman, of St. Louis, Mo., "On the Influence of Geological Formation on the Character of Disease."

20. Dr. Henry M. Builitt, of Louisville, Ky., "On the Use and Effect of Applications of Nitrate of Silver to the Throat, either in Local or General Disease."

21. Dr. Robert F. Campbell, of Augusta, Ga., "On the Pathogenic Influence of Feather Beds."

22. Dr. James Bolton, of Richmond, Va., "On the Administration of Anæsthetic Agents during Parturition."

23. Dr. Henry Taylor, of Mount Clements, Mich., "On Dysentery."

24. Dr. F. Donaldson, of Baltimore, Md., "On the Present and Prospective Value of the Microscope in Disease."

25. Dr. R. L. Howard, of Columbus, Ohio, "On the Pathology and Treatment of Scrofula."

*Committee on Plans of Organization for State and County Societies.*—Isaac Hays, M. D., of Pennsylvania, Chairman; Worthington Hooker, M. D., of Connecticut; Josiah Andrews, M. D., of Michigan; B. R. Wellford, M. D., of Virginia; A. L. Pierson, M. D., of Massachusetts.

*Committee on Medical Literature.*—T. S. Bell, M. D., of Kentucky, Chairman; Samuel H. Pennington, M. D., of New Jersey; Ed. H. Parker, M. D., of New Hampshire; William K. Bowling, M. D., of Tennessee; Zina Pitcher, M. D., of Michigan.

*Committee on Medical Education.*—B. R. Wellford, M. D., of Virginia, Chairman; Resign Lowe, M. D., of Iowa; Lyndon A. Smith, M. D., of New-Jersey; Jacob Bigelow, M. D., of Massachusetts; L. A. Dugas, M. D., of Georgia.

*Committee on Volunteer Communications.*—Drs. C. A. Pope, Thos. Reyburn, John S. Moore, J. B. Johnson and A. Litton, of St. Louis, Mo.

*Committee of Arrangements.*—Drs. J. R. Washington, J. S. Moore, S. Pollok, Thos. Reyburn, J. O'Farrar, W. M. M'Pheeters, C. W. Hempstead and E. S. Lemoine, of St. Louis, Mo.

*Committee on Publications.*—Dr. D. F. Condie, of Pennsylvania, Chairman; Dr. E. L. Beadle, of New York; Dr. A. Stillé, Pennsylvania; Dr. I. Hays, Pennsylvania; Dr. E. S. Lemoine, of Missouri; Dr. G. Emerson, Pennsylvania; Dr. G. W. Norris, Pennsylvania.

On motion of Dr. Watson, of New York, the name of the Committee on Volunteer Communications was changed to that of Committee on Prize Essays.

Dr. Wellford resigned his place as Chairman of Committee on Medical Education, and the President was authorized to fill the vacancy.

